# Alcohol use, self-harm and suicide: a scoping review of its portrayal in the Sri Lankan literature

**DOI:** 10.1016/j.heliyon.2023.e17566

**Published:** 2023-06-25

**Authors:** Jane Brandt Sørensen, Melissa Pearson, Janaka Pushpakumara, Dominique Leth-Sørensen, Alexandra Buhl, Flemming Konradsen, Lalith Senarathna

**Affiliations:** a Department of Public Health, University of Copenhagen, Øster Farimagsgade 5, Building 9, 1014, Copenhagen K, Denmark; b Central Clinical School, Faculty of Medicine and Health, The University of Sydney, Australia; c Department of Family Medicine, Faculty of Medicine and Allied Sciences, Rajarata University of Sri Lanka, Sri Lanka; d Department of Public Health, University of Copenhagen, Denmark; e Department of Health Promotion, Faculty of Applied Sciences, Rajarata University of Sri Lanka, Sri Lanka

**Keywords:** Suicide, Self-harm, Alcohol, Sri Lanka, Scoping review

## Abstract

**Background:**

Suicide is a global public health problem. Compared to other middle-income countries, much literature has been generated on the topic of self-harm and suicide in Sri Lanka. Harmful use of alcohol is a well-known risk factor to self-harm and suicide, however the connection needed further exploration.

**Aim:**

The aim was to investigate alcohol's role in self-harm and suicide in Sri Lanka to inform policy and prevention programs and future research priorities.

**Methods:**

We performed a scoping review exploring how the association between alcohol use, self-harm and suicide in Sri Lanka is presented in scientific literature from August 1, 2008 to December 31, 2022. Thematic analysis was used to explore emerging themes.

**Results:**

Altogether 116 peer-reviewed articles were included. Three themes emerged: (i) gendered, inter-relational explanations of alcohol's role in self-harm, (ii) hospital management of patients who co-ingested alcohol and pesticides, and (iii) proposed research and interventions targeting alcohol, self-harm and suicide. The articles' recommendations for policy, prevention and research priorities included: Family- and community-based alcohol, self-harm and suicide reduction interventions; viewing self-harm as a window of opportunity for health personnel to intervene in families affected by harmful alcohol consumption; and introduction of and increased access to treatment of alcohol use disorder at the individual level.

**Conclusion:**

Suggestions for alcohol, self-harm, and suicide prevention interventions were primarily targeted at the community, though this might also reflect the limited treatment, mental health, and alcohol support available in the country. Future research should explore and test context-appropriate interventions integrating alcohol and self-harm prevention and treatment.

## 1 Introduction

Self-harm and suicide are major global health problems. Approximately 39% of global deaths from suicide occurs in the WHO region of South-East Asia [1]. For each death, there are many more cases of self-harm [1]. We here use [2] to describe any deliberate injury to oneself with a non-fatal outcome, irrespective of intent of death. It thus captures how most cases of self-harm in, for example, the Sri Lankan context, are non-fatal and with little or no intent to die.

A risk factor globally recognized as associated with suicidal behaviour is the harmful use of alcohol [[1], [3], [4]]. The context of alcohol use before, during, and after self-harm and suicide is complex and varies globally and within countries [5]. Pesticides are a common means of self-harm and suicide in, especially, many Asian communities. Co-ingestion of alcohol and pesticides makes medical treatment difficult [6,7]. Further, the combination of pesticides and alcohol is associated with ingestion of larger quantities of pesticides and worse clinical outcomes [8,9].

Sri Lanka, a middle-income country, had one of the highest suicide rates worldwide in the 1990s [10]. This rate has since declined, primarily due to means restriction of the most toxic pesticides [11]. However, self-harm and suicide continue to be significant public health problems in Sri Lanka [12].

The harmful use of alcohol has numerous socio-economic impacts in Sri Lanka, such as domestic violence [13,14] and increased poverty [15,16]. Further, harmful alcohol consumption is associated with self-harm and suicide - either in the person who consumes the alcohol or in someone affected by it [[17], [18], [19], [20]]. Though alcohol consumption is relatively low in Sri Lanka, it is projected to increase to 5.2 L of pure alcohol for adults over 15 years by 2025, compared to 4.3 L in 2016 [21]. While these numbers include an estimate of illicit alcohol, it is difficult to get an accurate assessment as illicit alcohol is widely consumed. There are many abstainers in Sri Lanka and female drinking is rare [22]. Thus, those who do drink alcohol do so at much higher levels than official population average indicates.

Sri Lanka is one of a few middle-income countries where diverse self-harm and suicide literature exists, including a review of the available literature conducted by Pearson *et* al in 2014 [23]. Yet, a detailed account of the interaction between alcohol, self-harm and suicide has not been fully explored. Exploring existing scientific literature, the aim of this study was to investigate alcohol's role in self-harm and suicide in Sri Lanka to inform policy and prevention programs and future research priorities.

## 2 Methods

Since a scoping review of literature is relevant to identify knowledge gaps, scope a body of literature and to make recommendations for future research [24], this was deemed an appropriate tool to capture the available literature on self-harm, suicide, and alcohol use in Sri Lanka. We followed recommendations for conducting a scoping review [24] and the PRISMA framework [25] to ensure methodological rigor.

### 2.1 Inclusion and exclusion criteria

Peer-reviewed articles, editorials, commentaries, academic book chapters, and theses included in the study were: (i) fully accessible; (ii) published in English (iii) between August 1, 2008 and December 31, 2022 and (iv) addressed alcohol use in the context of suicide or self-harm in Sri Lanka. Research focusing on Sri Lankans living outside of Sri Lanka and studies whose authors were from Sri Lanka, but the content not specific to the context of Sri Lanka were excluded. Studies discussing terrorism-related suicide bombs and abstracts or corrections to papers were excluded. Information from newspaper articles were not included, given difficulty of ensuring their credibility.

### 2.2 Search strategy

The search for literature included the following steps [24]:

(i) An initial search for relevant databases and identification of key words.
(ii) Searches with key words that entailed a combination of terms related to Sri Lanka, pesticides (a common means of self-harm in Sri Lanka), self-harm, and suicide were used for each database (PubMed, Scopus, Web of Science, PsycINFO and Sociological Abstracts) for literature on suicide and self-harm in Sri Lanka. Hand searches were conducted through Google and Google Scholar.
(iii) Searches for additional studies in the reference lists of the identified reports were conducted.
(iv) Included literature were screened for whether they included a focus on alcohol's role in self-harm and/or suicide.

Two reviewers (JBS, DLS) reviewed the selected studies according to the inclusion criteria.

### 2.3 Synthesis

Included articles were uploaded to NVivo12 (QSR International) [26], where data was coded and organized to identify emerging themes, using thematic analysis. Study characteristics, such as publication date and study design, were coded and quantified. In the results section, references are specified where they are specifically illustrative of the point made. Findings are primarily based on studies where the self-harm, suicide and alcohol association is discussed in-depth. Included articles are therefore not represented equally.

## 3 Results

### 3.1 Study selection and characteristics

**Fig. 1:**
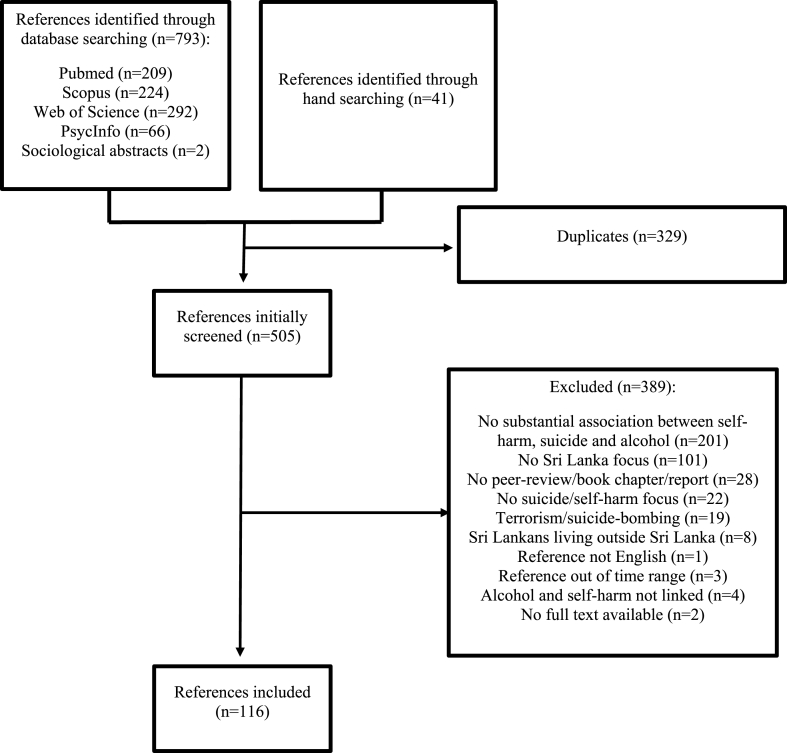
Selection process.

Searches generated 834 references. After removing 329 duplicates, 505 articles were screened. A total of 389 articles were excluded. This yielded 116 references eligible to be included in the study. Overview of articles included in the study is provided in Fig. 1 and the studies’ methodologies are specified in Table 1.

**Table 1:** Literature on the association between suicide and alcohol in Sri Lanka (2008–2022): breakdown of study types.

Study type	*n*	%
Experimental		
- Systematic review	4	3,4
- Randomized Control Trial (RCT)	8	6,9
- Case control	7	6,0
- Cohort	17	14,7
Descriptive quantitative	38	32,8
Qualitative	22	19,0
Expert opinion	20	17,2
**Total**	116	

### 3.2 Synthesis of results

Of the 116 articles, 8 had both alcohol and self-harm or suicide in the title. In 55 articles, the authors mentioned alcohol when explaining the background or rationale of the study. The three main themes emerging from the analysis included (i) gendered, inter-relational explanations of alcohol's role in self-harm and suicide, (ii) hospital management of patients who co-ingested alcohol and pesticides, and (iii) proposed research and interventions targeting alcohol, self-harm and suicide.

### 3.3 Gendered, inter-relational explanations of alcohol drinking, self-harm and suicide

#### 3.3.1 Age, gender and vulnerability to alcohol and subsequent self-harm or suicide

Across articles, alcohol drinking was explained to be unusual amongst women [27]. A study exploring demographic and clinical data of acute poisoning cases found that of the 243 individuals who reported to hospital after ingestion of profenofos (type of pesticide), 64 men (26.3%) had also consumed alcohol [7]. Different age groups of (male) drinkers were referred to be particularly vulnerable to consuming alcohol and subsequently self-harm. This included young men [20,28,29]. In a study of student perspectives of suicide, young men explained that consuming alcohol in a harmful manner often resulted in violent behaviour. Participants also explained how young men self-poisoned because they could not afford to financially support their alcohol consumption habits [30]. Older men were found to be a vulnerable group to alcohol, depression and self-harm in a study by De Silva [31] and alcohol co-ingestion and older age were associated with worse hospital outcome in patients who self-harmed in the study by Dhanarisi *et* al. [7].

#### 3.3.2 Men's alcohol consumption and its effect on women and children's self-harm and suicide

Women were commonly mentioned to be affected by someone else's alcohol use, typically a husband or a father's, which subsequently resulted in self-harm or suicide [19,[32], [33], [34], [35], [36], [37]]. Interpersonal conflict related to alcohol use was a predominant theme [32,36,[38], [39], [40], [41], [42], [43]], and were often related to struggles over socio-economic issues [19,44,45]. For example, women's refusal to hand over money for alcohol to their husbands often led to conflicts resulting in suicidal behaviour [37,41].

Knipe *et* al. found that the risk of attempted suicide nearly doubled in women living in households with someone who misused alcohol [46]. Furthermore, a cohort study by Fernando *et* al. of 22,000 individuals between the age of 12–18 found that factors associated with higher rates of self-harm included being female, no longer being involved in education, and living in a household with an individual with problematic alcohol consumption [47]. A social autopsy study of 15 maternal deaths showed suicide to be a common cause of death (4/15) and how poverty, social isolation, and poor social support in combination with alcohol use and violence lead to suicides [48]. In a study of 1000 antenatal women, Palfreyman showed that a husband's alcohol use and a woman's perception of alcohol problem drinking in a spouse were associated with depressive outcomes [49]. In a qualitative study Palfreyman further highlighted how Public Health Midwives stated that the primary risk for a woman's self-harm was her husband [50], noting how volatile relationships, violence and tension in the relationship - and subsequent self-harm or suicide - was linked to men's alcohol and drug abuse [50]. Widger, in an ethnographic study, highlighted a 45-year-old house-wife who self-harmed after being subject to violent abuse by her drunken husband [37]. In their qualitative study, Sørensen *et* al. explained how women resorted to self-harm to "teach him a lesson" and thereby hoped to change their husbands' alcohol behaviour. Due to gendered hierarchy, it was difficult for the women to be heard in other ways [35,41]. Marecek and Senadheera also found that women's self-harm seemed to improve their situation afterwards [36].

A qualitative study examining students' perspectives on suicidal behaviour showed how problems between parents and youth were often due to parental, most likely fathers', alcohol abuse. The harmful use of alcohol put families under strain, especially because parents would fight in front of children, making it difficult for them to get on with their studies [30]. Several articles explained how children and adolescents engaged in self-harming behaviour to stop a conflict between their parents [36,39]. Abeyasekera and Marecek highlighted how a young woman who felt “angry and sad” about her father's alcohol drinking, consumed paracetamol to compel her father to live up to his obligations of upholding his and the family's standing [35]. In an ethnographic study of suicide in a rural village, Widger noted how children were playing ‘families’, which entailed a drunk, shouting father. The children adopted the roles of the parents' and played out self-harm to stop the fighting [39].

#### 3.3.3 Women being blamed for others’ self-harm

Several articles highlighted how women's perceived inappropriate behaviour was linked to others' self-harm [41,51]. Both Widger and Sørensen *et.* al noted how women's migration to improve their families' economy and sometimes leave a difficult marriage was connected to alcohol and self-harm [41,51]. Widger outlined how it was commonly believed that men spend their wives' remittances on alcohol [51]. Husbands of migrating women were often perceived alcoholics, whether true or not, leaving them and children in a difficult situation. At the same time, wives and mothers migrating were commonly blamed for any misfortune, including self-harm and suicide [51]. For example, in Widger's ethnographic study, a young man's alcohol intoxicated suicide was explained by mourners to be due to his mother's migration for work. Without his mother present he followed the wrong path in life, which cost him his life [45].

### 3.4 Hospital management of self-harm patients

In total, 27 clinically focused articles were concerned with treatment of individuals who co-ingested alcohol and pesticides [7,[52], [53], [54], [55]]. Here, it was discussed how co-ingestion with alcohol could influence the amount of pesticides ingested. In a qualitative study exploring rural doctors’ experience of managing self-poisoning patients, doctors were challenged in identifying patients who had ingested dangerous amounts of poison. This was especially difficult when patients had also consumed alcohol and subsequently expectorated most of the poison [53]. Dhanarisi *et* al. found that patients who co-ingested alcohol and organophosphorus insecticide required intubation more often and had a higher risk of death than those who did not co-ingest alcohol. The authors speculated whether this was due to larger quantities of pesticide ingestions by alcohol intoxicated individuals compared to individuals who had not consumed alcohol [7]. With a health system perspective, Sariola and Simpson ethnographically explored self-poisoning admissions to rural hospitals and ethical dilemmas in enrolling them in research [56]. These studies highlighted the challenges for medical and research staff when alcohol is part of the self-harm episode.

#### 3.4.1 Proposed interventions targeting alcohol, self-harm and suicide

A total of 8 articles stated how interventions targeting problematic alcohol use was needed in order to prevent self-harm, though without expounding on what they would entail [20,23,42,46,[57], [58], [59], [60], [61]]. Other articles suggested interventions and policies to target harmful alcohol consumption at the individual, community, or population levels to indirectly reduce self-harm and suicide (see Table 2).

**Table 2:** Recommendations for interventions targeting the harmful use of alcohol, self-harm and suicide.

Individual-based intervention strategies	Hospital-based intervention strategies	Community-based intervention strategies	Population-based intervention strategies
• Psychological treatment programs for heavy alcohol users [54] • Targeted brief interventions and treatment for alcohol problems [30,[62], [63], [64]]	• Education for health staff about ‘red flags’ to identify high risk persons presenting with self-harm i.e. those with alcohol use disorder [65] • Screening for AUD in individuals who have self-harmed [62]	• Advocacy programs • Waiting times for purchasing pesticides at vendor shops, particularly targeting customers who consumed alcohol [66,67] • Educational programmes about prevention of the harmful use of alcohol and drugs for school-children [68,69] • Community-based interventions to reduce alcohol consumption [27,40,41,65,68,[70], [71], [72], [73]]	• Taxes and policies such as ban on alcohol and tobacco advertising • Regulation of access to alcohol, especially through targeting illicit alcohol [27] • Campaigns against alcohol consumption [7,69] • Recognition of alcoholism as a disease [63] • Addressing problem alcohol use in national suicide and violence prevention policies [47]

### 3.5 Proposed research targeting alcohol, self-harm and suicide

The included articles emphasized several future research areas focusing on alcohol's role in self-harm. This included recommendations for more systematic research using epidemiological tools; devising methods to get more accurate estimations of alcohol consumption, including illicit alcohol; as well as the development of a culturally appropriate national survey providing policy makers with an accurate picture of alcohol consumption [20]. Testing of brief educational interventions appropriate for resource-poor settings targeting subgroups of individuals who have self-harmed, for instance individuals with alcohol problems, was also mentioned as a point for further research [74]. Eddleston *et* al. called for larger, clinical studies to comprehensively assess the interaction of acute and chronic alcohol use on outcome in poisoning [52]. The need for qualitative research was highlighted, specifically: exploring areas with higher levels of deprivation and problem alcohol use and the connection to higher levels of attempted suicide [46]; contextual factors such as dynamics within the marriage, gender inequalities and alcohol norms [41]; and illicit alcohol and barriers to its reduction [20].

## 4 Discussion

In this scoping review of 116 articles, we found alcohol discourses depicted in the Sri Lankan suicide literature both quantitatively, for example, through analysis of police and hospital data, as well as qualitatively via, for example, ethnographic investigations.

Inter-relational explanations of alcohol's role in self-harm were a common theme, which reflects the importance of relationships in Sri Lanka. In contrast to many high income settings, the self is embedded in a web of relations in Sri Lanka, and the individual is thus less important [35], which also explains why self-harm in Sri Lanka is typically not perceived to be about the individual [35]. The collective is also important in alcohol consumption in Sri Lanka, which is a social activity where men's participation is expected [75]. A qualitative study exploring alcohol consumption in Sri Lanka found that appropriate consumption was defined as ‘moderate consumption’ in covert, social and contained settings, and thus difficult to refrain from for men [58]. However, as this study points to, when men consume alcohol it not only affects themselves, but entire web of relations. Thus, self-harm and alcohol need to be viewed through familiar relations and collective identities.

The importance of relationships might also explain why suggestions for alcohol, self-harm and suicide prevention interventions are primarily targeted at the community. It should however be noted that the few individually targeted interventions suggested in the included literature might also reflect the limited treatment, mental health and alcohol support available in the country, especially in rural areas.

Several issues were not mentioned in the literature included for this study. The connection between depression and alcohol use, as evidenced consistently in international literature [3], was scarcely mentioned in the included articles. This may indicate inefficiencies in Sri Lanka's mental health information infrastructure in addition to recognition of such mental illnesses and comorbidities by medical staff. Only one source remarked the role of religion, suicide and alcohol [76]. There were no studies on national cost of alcohol where self-harm featured as a cost. No articles focused on the role tourism plays in introducing new types and patterns of alcohol.

This review highlighted how self-harm and suicide is contextual and complex, without a one-sided cause.Considering how both alcoholconsumption and self-harm happens within and have consequences for web of relations, family- and community-based interventions seem most relevant in the Sri Lankan context. Future research should explore and test context-appropriate interventions targeting this, preferably through participatory methods, thereby including the voices of a hard-to-reach population group, such as harmful alcohol consumers. Cases of self-harm provide a window of opportunity for health personnel to identify and intervene in families affected by harmful alcohol consumption. At the individual level, introduction of and increased access to treatment of alcohol use disorder appear appropriate. Qualitative studies based on verbal autopsy would be useful to further explore the link between alcohol, self-harm and suicide.

### 4.1 Limitations

We only included literature in English, though other conclusions could have been captured in Sinhala and Tamil literature. We conducted a scoping review, acknowledging that a systematic review with quality ratings of the included literature would have equipped us to make more robust conclusions.

## 5 Conclusion

Alcohol consumption is an important part of many cases of self-harm and suicide. The findings from this scoping review of the association between alcohol use, self-harm and suicide in Sri Lanka included three main themes: i) gendered, inter-relational explanations of alcohol's role in self-harm, (ii) hospital management of patients who co-ingested alcohol and pesticides, and (iii) proposed research and interventions targeting alcohol, self-harm and suicide. Suggestions for alcohol, self-harm and suicide interventions were primarily targeted at the community, which might reflect the limited treatment, mental health and alcohol support available in Sri Lanka. Future research should explore and test context-appropriate interventions integrating alcohol and self-harm into prevention and treatment efforts.

## Author contribution statement

All authors listed have significantly contributed to the development and the writing of this article.

## Data availability statement

Data will be made available on request.

## Declaration of competing interest

There are no conflicts of interest.
